# Ambient Artificial Intelligence Scribes: A Pilot Survey of Perspectives on the Utility and Documentation Burden in Palliative Medicine

**DOI:** 10.3390/healthcare13172118

**Published:** 2025-08-26

**Authors:** James Patterson, Maya Kovacs, Caitlin Lees

**Affiliations:** 1 Palliative Care Family Medicine Enhanced Skills, Department of Family Medicine, Faculty of Medicine, Dalhousie University, Halifax, NS B3H 2Y9, Canada; 2 Division of Palliative Medicine, Dalhousie University, Halifax, NS B3H 2Y9, Canada; caitlins.lees@nshealth.ca

**Keywords:** artificial intelligence (AI), ambient scribe, palliative medicine

## Abstract

Background/Objectives: There is growing evidence to support ambient artificial intelligence (AI) scribes in healthcare to improve medical documentation by generating timely and comprehensive notes. Using the Plan–Do–Study–Act (PDSA) methodology, this study evaluated the utility and potential time savings of an ambient AI scribe, Scribeberry, (V2), in a palliative medicine outpatient setting, comparing it to the standard practice of dictation. Methods: This prospective quality improvement study was conducted at an academic medical center by two palliative medicine resident physicians. Residents documented patient visits using a freely available ambient AI scribe software program, Scribeberry, as well as using standard dictation software. Primary outcome measures included the editing time for the AI scribe and the dictating and editing times for a dictated manuscript, as well as subjective assessments of the accuracy, organization, and overall usefulness of the AI-generated clinical letters. Results: A heterogenous response was seen with the implementation of an AI scribe. One resident saw a statistically significant reduction (*p* < 0.025) in the time spent on clinical documentation, while a second resident saw essentially no improvement. The resident who experienced time savings with the ambient AI scribe also demonstrated a significant improvement in the graded organization and usefulness of the AI outputs over time, while the other resident did not demonstrate significant improvements in any of the metrics assessed over the course of this project. Conclusions: This pilot study describes the use of an ambient AI scribe software program, Scribeberry, in the community palliative medicine context. Our results showed a mixed response with respect to time savings and improvements in the organization, accuracy, and overall clinical usefulness of the AI-generated notes over time. Given the small sample size and short study duration, this study is insufficiently powered to draw conclusions with respect to AI scribe benefits in real-world contexts.

## 1. Introduction

Documentation for the electronic health record plays a significant role in physician burnout, and generative artificial intelligence (AI) has been proposed as a potential solution to the burdensome documentation process [1]. Ambient AI scribes use language processing technologies to record clinical encounters and produce narrative summaries [2]. There is growing evidence to support the potential for AI to optimize healthcare workflows, reduce burnout, and increase physician workplace satisfaction. It has been suggested that AI scribes may improve medical documentation by generating timely and comprehensive notes. Large language models (LLMs) are a form of AI that is trained on data and can generate text and respond to requests, such as GPT-4 [1]. Ambient AI scribes are designed to use LLMs to generate clinical notes within minutes after a clinic visit [3,4]. In addition to improving documentation times, it has also been reported that ambient AI scribe software can benefit clinicians by recording difficult-to-remember details and bypassing the need to write notes from scratch, thereby decreasing the overall cognitive load [5].

These benefits are particularly relevant in the community palliative medicine context, where home visits pose an environmental and time challenge in completing documentation. There is a unique workflow required when conducting home visits for palliative patients, and AI may have a particularly useful role in this documentation process. While the documentation time varies among individual clinicians within each specialty, those practicing in primary care and medical specialties spend more than twice as much time on notes as surgical colleagues [6].

Although AI-generated clinical documentation aims to lessen the administrative burden, it may contain errors of omission or addition or nuanced inaccuracies (e.g., tone of voice, body language) and must always be reviewed [3,5]. The accuracy of ambient scribes depends on several factors, including the audio quality, medical terminologies, and the complexity of the clinical encounter [7]. Although AI-generated notes still need to be reviewed by the author, as with dictation software, current AI scribes can be trained via machine learning to produce real-time transcripts of encounters, as compared to the delayed dictation output. To fine-tune the output of ambient AI scribes, clinicians can use instructions, referred to as “prompts”, to optimize the process [8].

This quality improvement study aims to explore the time savings and utility of a freely accessible AI scribe program in a community palliative medicine service in Halifax, Nova Scotia, Canada. This small, pilot study will evaluate the time spent documenting using an ambient AI scribe and compare it to the current standard time required for the use of dictation software. We will also assess for improvements in the quality of the AI’s outputs over time, as users fine-tune their prompts to better meet their organization and stylistic objectives for documentation.

## 2. Materials and Methods

This quality improvement project was conducted by two residents completing subspecialty training in palliative medicine, based in Dalhousie University’s Queen Elizabeth II (QEII) Health Sciences Centre in Halifax, Nova Scotia, Canada. The local Research Ethics Board determined that the project did not require formal institutional review based on its classification as a quality improvement initiative. To evaluate the effectiveness of our intervention, this study used the Plan–Do–Study–Act (PDSA) methodology [9].

### 2.1. Study Planning

**Figure 1:**
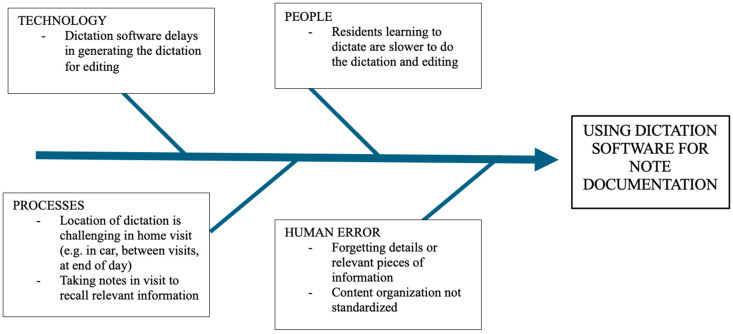
Fishbone diagram depicting the use of dictation software to document clinical encounters in the home visit setting for palliative medicine.

The process at the QEII Health Sciences Centre for clinical documentation in the community palliative medicine service was analyzed. The current standard practice at the time of writing involves dictating a clinical letter following a visit using dictation software, waiting for the letter to be transcribed, editing the transcript once available, and reviewing the letter before approving it and having it uploaded to the patient’s chart. There were a number of identified challenges associated with using the dictation software in the community context, as identified in the fishbone diagram in Figure 1. After reviewing multiple ambient AI scribe tools, “Scribeberry” was selected as the tool of choice for this project based on recommendations from the Nova Scotia Health Authority Research and Innovation Privacy team. Scribeberry notes are encrypted in transit, and Canadian data stay in a Canadian server. Scribeberry does not train models on user data. Audio is streamed to Scribeberry’s self-hosted transcription service with no permanent audio files. Data cannot be seen or repurposed by Scribeberry.

### 2.2. Use of Ambient AI Scribe

The AI scribe was piloted for two 1-month periods: the first from December 2024 to January 2025 and the second from February to March 2025. These time periods were selected on the basis of academic scheduling and were limited based on staff availability. Data were collected by a separate resident for each period. Each resident generated a base prompt for the software prior to use, which provided instructions for note organization, content, and style. This was based on the standard note structure used at our institution for palliative medicine consultations. Given the palliative medicine patient population seen in the home, verbal consent was chosen given the challenges with written consent for patients with limited function. Verbal consent was obtained from patients in all cases prior to using the ambient scribes in clinical encounters. After the encounter, the AI-generated note was reviewed and edited, and the time to complete this was then recorded. Each resident also dictated a clinical note for each visit and recorded the time spent dictating, as well as the time spent editing the dictated clinical letter.

### 2.3. Implementing the PDSA Methodology

**Figure 2:**
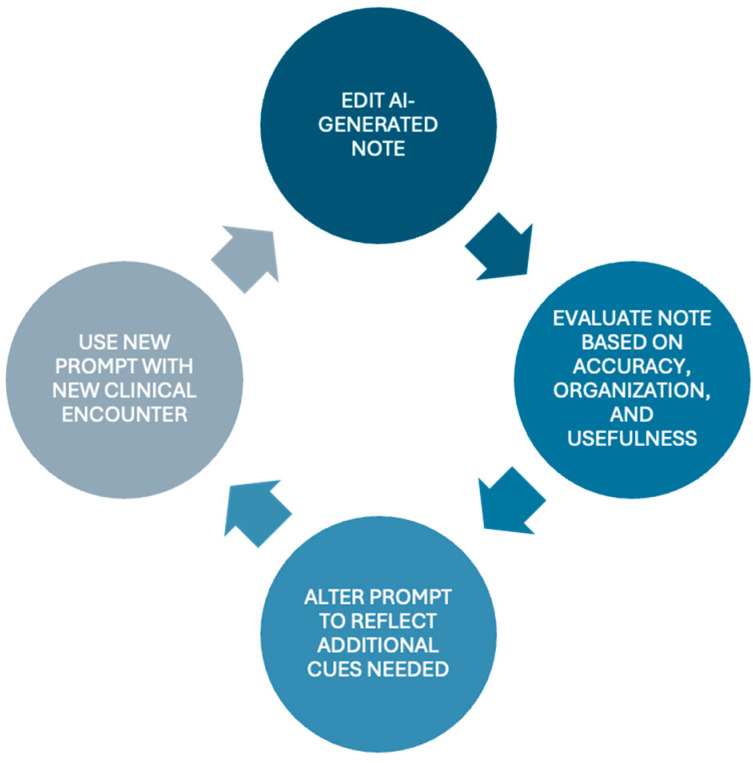
Plan–Do–Study–Act (PDSA) cycle used in this project to evaluate the outputs of the AI scribe after each clinical encounter. Resident physicians would review the AI-generated note and evaluate the output based on accuracy, organization, and usefulness. Then, based on the individual preferences of the resident, the prompt would be edited to improve the output for the next encounter so that it more accurately and comprehensively generated the desired clinical documentation.

Prior to editing each of the AI outputs, the residents evaluated the quality of these outputs using three metrics—“organization”, “accuracy”, and “usefulness”. This was performed using a Likert scale ranging from scores of 1 to 5, where 1 was “very poor”, 3 was “neutral”, and 5 was “excellent”. “Organization” reflected how well the AI structured its initial output, while “accuracy” measured the inclusion of accurate and relevant clinical information, as well as if there were any errors. “Usefulness” scored the AI on the degree to which the resident felt that the unedited output would be acceptable clinical documentation. This is a subjective concept that we included to represent the overall output as potential unedited clinical documentation to the individual user. Inter-rater reliability was not evaluated given that both resident physicians were not present at the same visits. Following this, as per the Plan–Do–Study–Act (PDSA) methodology used in this project, adjustments to the prompt were made to add, alter, or remove components to improve the generated outcome with the new clinical encounter. The alterations made were determined by the individual resident physician based on the generated AI output and their unique preferences and styles of clinical documentation. These prompts were adjusted following each clinical encounter. A graphical representation of this process is depicted in Figure 2.

### 2.4. Statistical Analysis

We used linear regression modeling to determine if there was a significant improvement in transcript grades of the three metrics—“organization”, “accuracy”, and “usefulness”—of the AI-generated clinical documentation over time. Separate models were created for each grade for each resident. Grades were considered to have significantly improved over time if the slope in the linear relationship between the transcript grade and number of iterations of scribe use was both positive and statistically significant from 0.

To determine if there was a significant reduction in the time required to document when using the AI ambient scribe over the course of the study period, we used a split plot analysis of variance (ANOVA) model. This was selected as it allowed for an analysis of variance context, and, for the purposes of this model, individual patients were considered as blocks, documentation type (either dictation or ambient scribe) was the subplot factor, and resident was considered the main plot factor. In the case of a significant interaction present in the ANOVA model, the mean time to document between the ambient scribe and dictation was compared using separate paired T-tests. In this case, a simple Bonferroni correction was used to account for the inflated error rate of conducting multiple individual T-tests. All data were checked to ensure that they satisfied the assumptions of normality for parametric tests. All analyses were conducted in R (version 4.4.2).

## 3. Results

### 3.1. Patient Consent and Scribe Success

**Figure 3:**
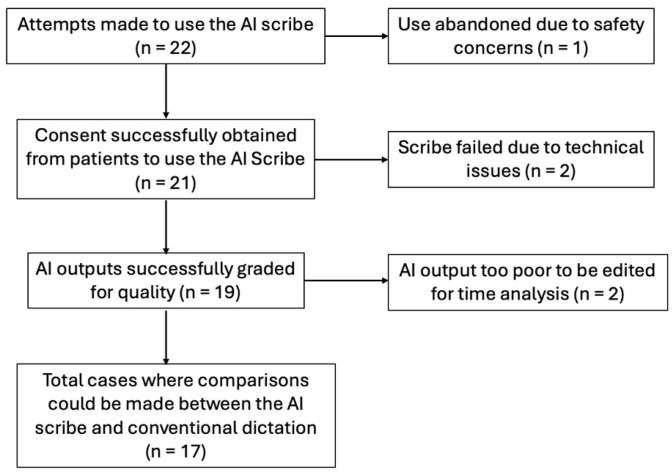
CONSORT-style diagram displaying patient consent and scribe success during the data collection process.

A total of 22 attempts were made to use the ambient scribe by the two residents across both study periods. In one case, the use of the scribe was abandoned for hygiene purposes. Consent was successfully obtained from patients to use the ambient scribe in the 21 remaining cases. In two of these cases, the scribe failed due to technical issues. This left a total of 19 cases that were successfully graded for quality: 11 cases from Resident 1 and 8 cases from Resident 2. In two further cases (each of the resident’s first successful transcriptions), the time required to edit the script was not recorded as the output required more than 60 min of editing to be of acceptable organization and accuracy, and, given time constraints, the editing of the script was abandoned. This resulted in 17 cases where comparisons could be performed regarding the time required to document with and without the ambient scribe: 10 cases from Resident 1 and 7 cases from Resident 2. The results are displayed in a CONSORT-style diagram in Figure 3.

### 3.2. Transcript Grades

**Figure 4:**
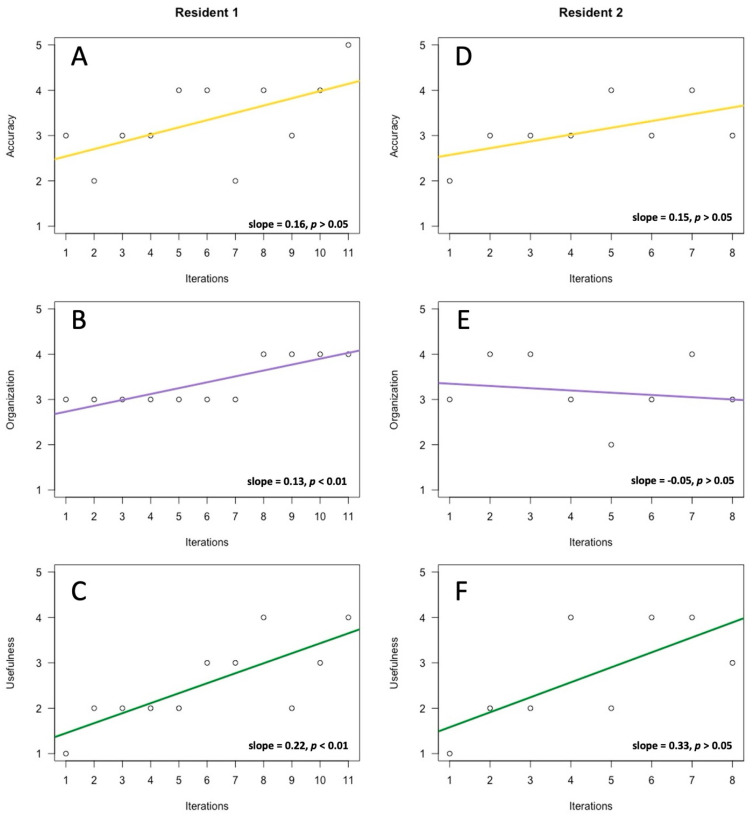
Results of the grading of the initial outputs of the ambient scribes. Each metric was graded on a Likert scale ranging from scores of 1 to 5, where 1 was “very poor”, 3 was “neutral”, and 5 was “excellent”. Results are graphed against the number of iterations of AI scribe documentation completed by each resident. Results show accuracy, organization, and usefulness for Resident 1 (**A**–**C**), as well as for Resident 2 (**D**–**F**). Solid lines depict the tread lines as determined by each linear model. Numeric values for slopes, as well as their statistical significance, are included for each.

Figure 4 displays the results of the grading of the AI scribe outputs via the metrics of “accuracy”, “organization”, and “usefulness”. Results are displayed separately for each resident. Of these, only organization (slope = 0.13, *p* < 0.01) and usefulness (slope = 0.22, *p* < 0.01) for Resident 1 displayed significant improvements over time. Other grades had positive slopes that were not statistically significant, with the exception of organization (slope = −0.05, *p* > 0.05) for Resident 2, which had a negative slope that was not statistically significant.

### 3.3. Documentation Time

**Figure 5:**
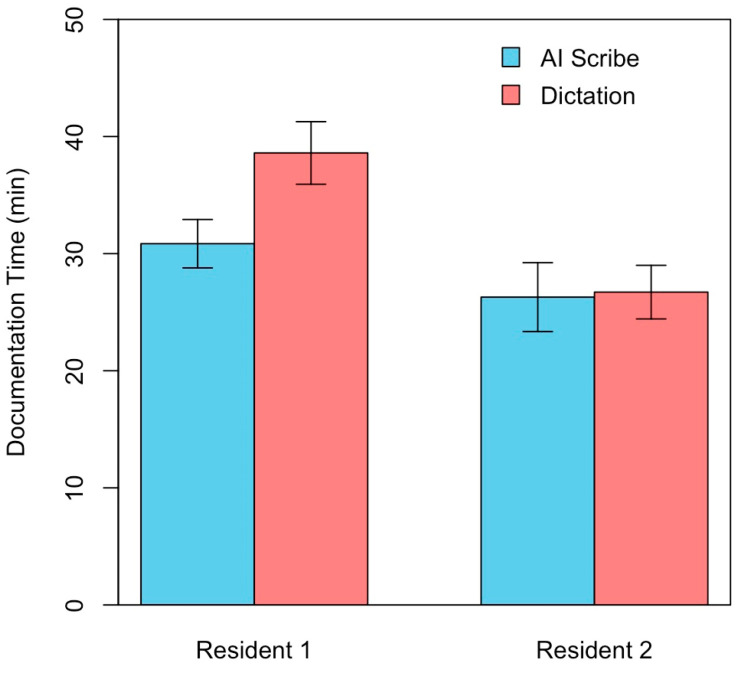
Comparison of mean documentation time in minutes between the ambient AI scribe and conventional dictation for each of the two residents involved. Error bars display 95% confidence intervals. Mean documentation time was significantly lower when using the AI scribe for Resident 1 (t = 3.55, *p* < 0.025), but there was no significant difference in the documentation time between the AI scribe and conventional dictation for Resident 2 (t = 0.23, *p* > 0.025).

Figure 5 displays the comparison of the mean documentation time using the ambient AI scribe compared to the use of dictation software. We used a split plot ANOVA model to determine if there was a significant difference in documentation time between the two methods across the two residents. However, this model revealed a significant interaction between the effects of the resident and documentation type on the documentation time (F-value = 5.70, *p* < 0.05). Therefore, we compared the documentation time to the documentation type for each resident separately using individual paired T-tests. For Resident 1, the mean documentation time was significantly lower for the AI scribe compared with dictation (t = 3.55, *p* < 0.025). However, for Resident 2, there was no significant difference in the documentation time between the AI scribe and dictation (t = 0.23, *p* > 0.025).

Overall, our study showed a mixed response with respect to time savings and improvements in note quality over time when using an ambient AI scribe. Resident 1 saw a statistically significant reduction in the time spent on clinical documentation, while Resident 2 saw no improvement. Resident 1, who experienced time savings, also demonstrated a significant improvement in the graded “organization” and “usefulness” of the AI outputs over time, while Resident 2 did not demonstrate significant improvements in any of the metrics assessed over the course of this project.

## 4. Discussion

Given the need for high quality and comprehensive clinical documentation in our current healthcare system, there is interest in identifying the most efficient documentation tools. The process for palliative medicine home visit clinical documentation at the QEII Health Sciences Centre in Halifax, Nova Scotia, Canada was evaluated, and the process was compared with the use of ambient AI scribe-generated clinical documentation.

There are several factors that may contribute to the different magnitudes of benefit experienced by individual scribe users with respect to time savings. Given the design of our pilot study, it was insufficiently powered to draw conclusions with respect to AI scribe benefits in real-world contexts. In our study, the resident that did not have statistically significant time savings had an overall lower documentation time with both dictation and ambient AI scribe use, as compared to the resident who did have significant time savings. This may suggest that a clinician’s efficiency baseline has an impact on the magnitude of benefit expected from introducing an AI documentation tool. Clinicians who are efficient dictators, due to experience, workflow, or varying contexts, may simply be less likely to see time savings when using an AI scribe. User hypervigilance and “repurposing” time spent writing into time spent editing [4] may also have an impact in this context. Our findings are consistent with other research demonstrating a mixed association of ambient AI scribe use with time savings [10,11,12].

Interestingly, it has been noted that “high users” of ambient AI scribe software, intuitively defined as those that use the software most frequently, were found to derive the most time savings per note as compared to “low” users [10]. This perhaps relates to a concept that has been described previously in the literature as “user phenotypes”, with certain users deriving more utility from ambient scribe technologies than others [1]. Individual users may show variations in the rate at which they are able to implement improvements to the outputs from AI scribes over time, as it is an individualized learning process. One contributing factor in our study may have been the overall short duration, which could have constrained the required time to establish comfort and familiarity with the software process and generate a robust prompt that would function in a variety of clinical encounters. Additionally, both of the resident physicians in this study did not have any previous experience with the use of AI scribe software. Tierney et al. have reported a “dose–response” relationship in AI scribe usage and benefit, identifying that the maximum benefit in time savings seems to be concentrated among the highest users [10]. In our study, it is possible that the resident who did not show improvements in the grades of scribe outputs would have done so if examined over a longer period, supported by the fact that this resident had a lower total number of patient visits recorded.

It has been suggested that clinicians using AI scribes should “view their new role as note editor” [2]. This shift in roles may not be as intuitive and simple as was initially proposed with the launch of numerous AI scribes. These clinical encounters are “coauthored narratives” and require cognitive work and perhaps even narrative and editorial training [2]. Training the ambient AI scribe gives the user the opportunity to create a set of instructions and customize this to their preferences. In this study, the individual residents began the work of “training” the scribe. In our review of the existing literature, it seems to be more common that AI scribes are procured as “trained by, and acquired from, an external vendor” [13]. This training was found to be a more labor-intensive process than initially anticipated—and, although it gives the clinician an opportunity to shape their future notes, it is an upfront cost with use of an ambient AI scribe to have it “fit” particular documentation styles and preferences. In our study, we were able to show evidence of improvements in the organization and overall clinical usefulness of the AI-generated notes, but not in the accuracy of the information the AI collected. The “training” of AI scribes by clinicians may be a process that helps with style and clarity rather than the accuracy of the information that the scribe detects, but our results must be interpreted with caution given the very small sample size.

There have been few peer-reviewed published studies to date on ambient AI documentation technology in clinical practice, and there is no published research, to our knowledge, on the applicability of ambient AI scribes in the community palliative medicine setting. It has been suggested that ambient AI scribes may enhance the physician–patient relationship by increasing the quantity and quality of time that physicians spend listening and engaging with their patients and reducing time on a computer [13]. This may be particularly relevant to palliative medicine, geriatric medicine, and oncology, given the importance of communication and the provider–patient relationship in end-of-life scenarios. The home visit environment of palliative medicine visits may also bring forth unique privacy considerations with respect to the use of ambient AI scribe software. Our institutional privacy office did not identify any additional components required for the consent or procedure for our pilot project; however, this may be institution-specific and should be a consideration for implementation at other sites.

Integration into existing workflows is another challenge identified with the use of ambient AI scribe technology. Currently, if ambient AI scribe software were to be used at our institution, the clinician would have to generate an “empty” dictation to then input the clinical documentation generated by the ambient AI scribe. Real-time transcription systems that directly integrate into EHR infrastructure would improve the workflow efficiency significantly [14]. Additionally, it can be difficult to integrate new technology into palliative medicine home visits, due to the unpredictable nature of providing care outside of conventional clinical spaces. Technological disruptions are also an important consideration in the home visit environment, given the inconsistent internet connectivity and ambient noise.

Prompt development and refinement is an area of particular interest in the use of ambient AI scribe software, and the use of standardized prompt templates or prompt libraries would likely be of particular benefit. Additionally, guidelines for generating and editing prompts should be created in the future through further research ascertaining the most effective prompt structure and content.

There is little known about the documentation accuracy, error type, and subsequent patient harm that may stem from ambient AI scribe use [15]. Evaluating the safety of AI-generated clinical documentation is likely to be of significant concern as the use of AI increases in the healthcare environment. We did not formally collect data on the frequency of omissions, additions, or incorrect outputs. Other limitations of this study include the small sample size, inter-patient complexity, and differences in the backgrounds of the two resident physicians contributing data. Given that this was a pilot study, the sample size was limited to the only two palliative care resident physicians during this academic year. Future research should involve larger, more diverse cohorts of clinicians, incorporate patient perspectives, and collect data on errors, additions, and omissions. Patient perception during the clinical encounter with the use of ambient AI scribe software in the palliative medicine context is likely to be a particularly important future direction of research, in addition to evaluations of providers’ perceptions. Ambient AI scribe software has the potential to be utilized in many clinical contexts, and further research addressing the individualized considerations for use in certain specialties will be important.

## 5. Conclusions

Ambient AI scribes have the potential to improve the clinical documentation efficiency in various medical contexts. This pilot study describes the use of an ambient AI scribe software program, Scribeberry, in the community palliative medicine context. Our results showed a mixed response with respect to time savings and improvements in the organization, accuracy, and overall clinical usefulness of the AI-generated notes over time. Given the small sample size and short study duration, this study is insufficiently powered to draw conclusions with respect to AI scribe benefits in real-world contexts. This paper aims to begin a conversation around the potential utility and limitations of using ambient AI scribe software in palliative medicine and broader contexts.

## Data Availability

The individual documentation time data presented in this study are available on request from the corresponding author. The raw and edited clinical documentation is not presented in this article due to privacy reasons, as it contains personal health information.

## Abbreviations

The following abbreviations are used in this manuscript:

AI	Artificial Intelligence
PDSA	Plan–Do–Study–Act
